# Knowledge and Communication About the Menstrual Cycle Among Rhythmic Gymnasts, Ballerinas, and Dancers

**DOI:** 10.3390/ijerph22010013

**Published:** 2024-12-26

**Authors:** Marina Schulz, Kari Bø, Marte Charlotte Dobbertin Gram

**Affiliations:** 1 Department of Sports Medicine, Norwegian School of Sport Sciences, 0806 Oslo, Norway; 2 Department of Obstetrics and Gynecology, Akershus University Hospital, 1478 Lørenskog, Norway

**Keywords:** aesthetic sports, communication in sports, education, female athlete, menstrual cycle, knowledge

## Abstract

While moderate exercise supports regular menstrual cycle (MC) function, many female athletes experience MC symptoms that negatively influence their training and performance. Hereby, knowledge and communication about this topic are important to promote an athlete’s health and wellbeing. Hence, this study aimed to assess the knowledge and communication surrounding the MC among Norwegian rhythmic gymnasts, ballerinas, and dancers. In total, 116 rhythmic gymnasts, ballerinas, and dancers aged ≥ 16 years training ≥ 3 days per week completed an electronic questionnaire. Of these, 63% (n = 73) reported a lack of knowledge about the MC’s influence on physical fitness and performance, and 39% (n = 45) rated their coaches’ or pedagogues’ knowledge as low. With only 32% of participants discussing the MC with their coaches/pedagogues, communication was found to be limited. Seventy-three percent felt uncomfortable during training or competition due to their attire and recommended darker colors and more options in size and layering. The study highlights a significant gap in knowledge and communication about the MC among female athletes and coaches/pedagogues in aesthetic sports. It also recommends improving athletes’ choice of attire for greater comfort and confidence.

## 1. Introduction

The menstrual cycle (MC) lasts approximately 21 to 35 days and is regulated by fluctuating levels of hormones such as estrogen and progesterone [1]. The MC is divided into four phases: the menstruation or bleeding phase, the follicular phase, the ovulation phase, and the luteal phase [2,3,4]. Disruptions to the MC can be caused by stress, weight loss, eating disorders, or high levels of exercise, resulting in conditions like amenorrhea (absence of menstruation), dysmenorrhea (painful periods), or heavy menstrual bleeding (HMB) (excessive or prolonged menstrual bleeding up to 80 mL or more) [5,6,7].

While moderate exercise seems to support a regular MC [8], a considerable number of female athletes still experience MC symptoms or even MC disorders, which can adversely affect their participation in sports and training [9]. Research on knowledge and communication about the MC among athletes has mainly focused on endurance and team sports. Studies have highlighted a lack of understanding about the MC’s impact on performance, as well as barriers to discussing MC-related matters [10,11,12,13,14]. For example, Solli et al. [10] found that female biathlon athletes lacked knowledge about the MC’s influence on their performance. Similarly, Laske et al. [14] indicated that there seems to be a deficit in communication regarding the MC among athletes and coaches. They surveyed 1195 female athletes from a range of team and individual sports, but the study did not provide specific details on the sports included. Von Rosen et al. [11] included 48 female athletes (4.4%) from aesthetic sports in their survey of 1086 participants, with only one rhythmic gymnast and no ballerinas or dancers. Taim et al. [13] surveyed 90 athletes from 14 different sports, including six athletes from gymnastics and ice skating, both of which are considered aesthetic sports. The principal findings of a study by Bergström et al. [12] showed that female football players, as well as their coaches, expressed limited knowledge about the MC’s impact on performance, highlighting a lack of communication.

Aesthetic sports, categorized as weight-sensitive by the International Olympic Committee (IOC) [15] place female athletes at higher risk of menstrual disorders due to the pressure for low body weight, specific aesthetic ideals, and strict dress codes, which also may contribute to discomfort during menstrual bleeding [16,17]. Despite these special challenges, evidence of knowledge and communication about the MC is sparse in the field of aesthetic sports. Therefore, the aim of this study was to examine the knowledge and communication about the MC among rhythmic gymnasts, ballerinas, and dancers, as well as their experiences of how their attire influences their performance and wellbeing.

## 2. Materials and Methods

### 2.1. Study Design

This study employed a cross-sectional design to analyze data from a survey conducted among Norwegian rhythmic gymnasts, ballerinas, and dancers.

The study was approved by the Regional Ethics Committee (REK sør-øst A 230565) and the Norwegian Centre for Research Data (NSD/SIKT 702729) and conducted in accordance with the principles of the Helsinki Declaration (World Medical Association, 2008). All participants gave written consent to participate.

### 2.2. Participants

Female rhythmic gymnasts from rhythmic gymnastics (RG) clubs that are members of the Norwegian Gymnastics Federation (NGTF), ballerinas from the Norwegian National Ballet, and dancers/dance students from several Norwegian dance institutions (Oslo National Academy of the Arts, Bårdar Academy, Kristiania University College, and Spin Off) were invited to participate. Purposeful sampling was employed to recruit participants, as this method allows for the selection of individuals who are particularly relevant to the research objectives [18]. To be eligible for participation in the study, participants were required to meet the following inclusion criteria: They had to be at least 16 years of age, engage in their chosen sport or dance at least three days per week, and demonstrate proficiency in either Norwegian or English.

### 2.3. Procedure

In October and November 2021, RG club head coaches and contact persons from the dance institutions in Oslo were invited to share information about the study and the survey link with eligible participants within their respective organizations. Due to COVID-19 restrictions, the original plan to conduct face-to-face meetings and provide on-site access to the online questionnaire in November and December 2021 was altered. Instead, the RG club head coaches and contact persons were asked to distribute the survey link to their gymnasts, ballerinas, and dancers. Monthly reminders were sent from November 2021 to June 2022 to encourage further dissemination of the link.

The online questionnaire closed on 30 June 2022.

### 2.4. Outcome Measures

The present study was part of a larger study reporting on menstrual function, hormonal contraceptive (HC) use, symptoms during different MC phases, and the MCs’ self-perceived influence on physical fitness and performance. Menstrual function was assessed by relevant parts of the validated questionnaire Low Energy Availability in Females Questionnaire (LEAF-Q) [19], while HC use, symptoms during the MC phases, and the MCs perceived influence on physical fitness and performance were assessed by questions selected from questionnaires previously used among female cross-country skiers and biathletes [10,20]. Minor adjustments for relevance to gymnasts, ballerinas, and dancers were made.

The primary outcome of the present study was knowledge and communication about the MC, assessed by questions selected from Solli et al.’s questionnaire [10]. In addition, to assure relevance to gymnasts, ballerinas, and dancers, an inquiry related to the attire (leotard/suit/costume), specifically its color and shape, was integrated into the survey (see Supplementary Materials for the whole questionnaire).

### 2.5. The Questionnaire

The questionnaire contained six sections: (1) Participant characteristics; (2) Menstrual function; (3) HC use; (4) Symptoms during MC phases; (5) MCs self-perceived influence on physical fitness and performance; and (6) Knowledge and communication about the MC (Supplementary Materials). All sections were gathered in an online questionnaire platform, SurveyXact (by Ramboll), which was used for both distribution and data collection. The questionnaire included multiple-choice questions, dichotomous questions, and scales ranging from 0 (no impact/not affected at all) to 10 (considerable impact/very much affected). Open-ended responses were required only for ‘Other’ options or brief explanations, with age, height, and weight also requested as open-ended responses. The questionnaire took approximately 15 to 20 min to complete.

The questionnaire was pilot-tested prior to the start on three colleagues with gymnastics/dance backgrounds and reviewed by a group of gynecologists from a university hospital’s Department of Obstetrics and Gynecology, with no further changes made.

The present study reports findings from sections 1 and 6, with a few questions from sections 2 and 3 included to present background data on menstrual function and HC use. Section 1, Section 2, Section 3, Section 4 and Section 5 will be thoroughly reported in a separate publication.

### 2.6. Statistical Analysis

Data were analyzed using IBM SPSS Statistics version 28.0.0.0 (190). Data were first tested for normality to ensure normal distribution. Descriptive statistics were then used to calculate means and standard deviations (SD) of continuous/numeric data, while statistical frequencies were used to analyze the distribution of categorical data. Open-ended questions were reviewed manually, and responses were categorized. Participants were divided into three groups (rhythmic gymnasts, ballerinas, and dancers) for analysis. As no significant differences were found between the groups, the results are presented collapsed for all participants.

## 3. Results

Of 340 rhythmic gymnasts, ballerinas, and dancers meeting the inclusion criteria, a total of 123 participants answered the questionnaire (response rate of 36%). Seven files were incomplete and removed from the analysis, resulting in a final sample of 116 participants (response rate of 34%).

### 3.1. Participant Characteristics

Participant characteristics are presented in Table 1. Participants had a normal mean BMI score, and the majority were enrolled in university programs. On average, participants had 10.7 years of experience as gymnasts, ballerinas, or dancers, and they trained 22.9 h per week.

### 3.2. Menstrual Function and Hormonal Contraceptive Use

Table 2 presents background data on menstrual function and HC use. All participants had already gone through menarche. Over half of the gymnasts, ballerinas, and dancers reported having a normal MC, while one fifth stated that they did not know whether their MC was normal. Almost half (41%) of the participants reported experiencing heavy menstrual bleeding. Of these, 77% reported a score of 5 or higher. Over half (60%) of the participants were not using HC.

### 3.3. Knowledge and Communication About the MC

Half of the gymnasts, ballerinas, and dancers monitored their MC, and the majority stated that they did not think they had enough knowledge about the influence of the MC on training and performance (Table 3).

Most participants did not actively acquire knowledge about the influence of the MC on training and performance but would like to learn more, especially through school, social media (Instagram, TikTok), webinars, or menstrual apps (Table 3). When discussing the MC in relation to training and performance, most participants tended to converse with other athletes or friends. Only a small proportion of the participants engaged in discussions with health personnel or their coaches/pedagogues, and about one-third did not talk about it at all (Table 3). The majority stated that they were unsure about the level of knowledge their coach/pedagogue had about the MC. It was perceived that communication with a female coach/pedagogue was easier than with a male (Table 3).

### 3.4. Attire

During menstrual bleeding, 73% of all the gymnasts, ballerinas, and dancers stated that they felt uncomfortable in their attire due to its shape and/or color. More than half of them (n = 47) were dancers. Twenty-nine of all the participants submitted one or more suggestions for future improvement, which can be divided into three categories:

1. Color of pants (n = 20): wish to have darker color and/or no white colors;
2. Sizing of pants (n = 9): wish not to have too tight and small pants, especially during the bleeding phase;
3. Covering/layering (n = 10): wish to have more coverage and/or the possibility to wear a skirt over the pants during the bleeding phase.

## 4. Discussion

The results suggested that there were deficiencies in the knowledge and communication surrounding the MC among rhythmic gymnasts, ballerinas, and dancers. Furthermore, discomfort related to attire was revealed, particularly during menstrual bleeding.

### 4.1. Knowledge About the MC and Its Impact on Training and Performance

While nearly half of the participants surveyed reported monitoring their MC, over 20% indicated that they were unsure as to whether they had regular MCs, suggesting that the monitoring process might not always lead to a clear understanding of one’s MC. This highlights the necessity for more precise menstrual monitoring, as emphasized by Bouchard et al. [21], who advocated for tools such as hormonal tracking and apps. These methods could enhance menstrual health literacy and assist individuals in a more comprehensive understanding of their cycles, thereby addressing the observed deficiencies and supporting the health and performance of this population. Over 60% self-reported a lack of knowledge regarding the MC, training, and performance. Similarly, van Rosen et al. [11] found that only 24% of female athletes rated their knowledge about the MC as good or very good. In a separate study, Solli et al. [10] found that only 8% of the 140 cross-country skiers and biathletes surveyed rated their knowledge about the MC as good. In addition to the findings of von Rosen et al. [11] and Solli et al. [10], a study by Michelekaki et al. [22] with recreational female athletes also highlighted a significant knowledge gap regarding the MC. The study revealed that over 70% of the 164 athletes were unaware of the different phases of the MC and the specific hormones involved. This finding reinforces the necessity for enhanced educational initiatives on the topic. Additionally, only a modest proportion of participants in our study proactively sought this knowledge, but nearly all expressed a desire to pursue further education in the future. This confers with the study of Laske et al. [14] where 90% of the 1195 athletes from both team and individual sports expressed interest in learning more about the relationship between the MC and performance. The participants in the present study indicated that the provision of such information could be facilitated through social media, educational institutions, or webinars. Social media has previously been successfully used in other areas of women’s health to increase awareness and educate on sensitive topics. For instance, the Pink Protest utilized platforms like Instagram to mobilize thousands of young activists and raise awareness about menstrual health. Their #Free Periods campaign lead to tangible policy changes, such as increased access to menstrual products in schools in the UK [23,24].

In terms of the coaches’/pedagogues’ knowledge, 18% of the participants in our study indicated that they considered this to be sufficient. This finding is consistent with the results of Solli et al. [10], who found that 15% of the respondents believed that their coaches lacked adequate knowledge in this area. While Solli’s study focused exclusively on endurance athletes, our study focused exclusively on gymnasts, ballerinas, and dancers, thereby providing new information on these sport and dance populations.

In von Rosen’s study, over half of the respondents indicated that their coaches lacked sufficient knowledge about the MC. Furthermore, only 48 (4%) out of 1086 survey participants in van Rosen et al.’s survey were from the field of aesthetic sports, with only one rhythmic gymnast and no ballerinas or dancers. Additionally, Brown and Knight [25] found that not only did a significant number of female athletes report a lack of knowledge about the MC among their coaches, but coaches themselves also acknowledged a need for further education and training in this area, recognizing their own lack of preparation to support athletes. The study included a total of 14 participants, comprising seven athletes and seven coaches. However, no athletes from aesthetic sports were included, and only one coach from gymnastics participated.

### 4.2. Communication About the MC

A significant proportion of participants (32%) did not discuss the MC in the context of training and performance. As Laske et al. [14] and McHaffie et al. [26] demonstrated, such communication would be beneficial for all female athletes, as it can enhance overall well-being and performance. A paucity of communication and an inadequacy of education may contribute to the prevalence of untreated menstrual disorders [26]. Also according to McGawley et al. [27], effective communication is a crucial factor in improving menstrual health literacy. A lack of menstrual health literacy among practitioners, including inadequate knowledge and/or communication skills, could restrict the scope and quality of support that can be offered to female athletes and their coaches, thereby jeopardizing the long-term health of athletes. A deficiency in communication inevitably results in a corresponding deficit in menstrual health literacy [27]. In the present study, among the participants who discussed MC issues in relation to training and performance, only 12% identified their coach/pedagogue as a point of contact. Comparable studies by von Rosen et al. [11] and Solli et al. [10] showed similar results. In von Rosen’s study, only 11% of the surveyed athletes discussed the topic of “health of female athletes” with their coaches. Solli et al. [10] found that 27% of biathletes and cross-country skiers discussed MC issues with their coaches, a slightly higher number than among our participants. The athletes in Solli’s study exhibited a slightly higher mean age compared to the participants in our study, as the minimum age for inclusion among the skiers was 18 years, whereas the minimum age in our study was 16 years. This discrepancy in age may help explain why athletes in Solli’s study were more inclined to discuss MC issues, as younger women are generally less likely to engage in conversations about the MC [26].

More than 90% of our participants indicated a preference for communication with female coaches/pedagogues, a finding that aligns with those of other studies in different sports [10,14]. In endurance sports, most coaches are male, which may create obstacles to communication about the MC [10,11,26,28]. Levi et al. [29] identified the underrepresentation of female coaches as a pervasive issue. Research indicated that male coaches frequently regard communication about the MC as less crucial and feel less at ease addressing this topic than their female counterparts [30]. Kovács et al. [31] indicated that rhythmic gymnasts are frequently coached by women, which may contribute to reducing obstacles to discussing MC issues. However, since our study did not collect information on the gender of the coaches or pedagogues, we cannot determine whether this factor influenced the level of communication about the MC.

Furthermore, a lack of knowledge can reinforce several barriers, such as non-communication about the MC [13]. According to Bruinvels et al. [9], it is essential for coaches to understand the role of the MC and its effects on training and performance to effectively support their female athletes.

### 4.3. Attire

There is a paucity of research examining the well-being of female aesthetic sport athletes and dancers during menstrual bleeding in relation to their attire. Most participants in our study perceived that their attire had a negative impact on their ability to manage their MC. Light-colored pants and tight-fitting suits emerged as significant contributing factors. The dancers and ballerinas, in particular, reported feelings of discomfort due to the shape and/or color of their attire. Today, rhythmic gymnasts have the option to choose the color of their attire and to wear long tights or tight-fitting full-length leotards. Also, skirts are permitted, offering flexibility in their attire [32]. In contrast, dancers and ballerinas in group performances are constrained by the choreography and the choreographer’s vision, leaving them with less choice and may make it difficult to voice preferences out of fear of not being selected to perform.

A recent study revealed that female soccer players in white shorts exhibited diminished performance compared to those in non-white shorts, potentially due to discomfort associated with menstrual bleeding [33]. This finding is consistent with those reported in other sports, including rugby, artistic gymnastics, and judo, where the choice of attire, such as white shorts or leotards, has been linked to lower self-esteem and performance due to concerns about visible blood [34,35].

In addition, concerns have been raised regarding the visibility of tampon strings in tight or short outfits [36] and trampoline gymnasts have reported discomfort with their leotards during menstruation. This is due to the design of the garment limiting the options for carrying out personal hygiene, and judges have been known to enforce dress code rules despite the athletes’ discomfort [37]. Although none of the participants of our study indicated a desire to withdraw from competitions, many reported that their well-being was negatively affected by their attire during menstrual bleeding. This may have a negative influence on their performance. The participants of the present study recommended alterations to the demands regarding clothing, including the possibility of using darker colors, looser fits, and different layering options to enhance comfort and performance. These recommendations underscore the necessity of modifying dress codes to prioritize the well-being of female athletes.

### 4.4. Strengths and Limitations

To the best of our knowledge, this is the first study to specifically investigate the relationship between knowledge and communication about the MC within aesthetic sports such as rhythmic gymnastics, ballet, and dance. In total 116 gymnasts, ballerinas, and dancers completed the questionnaire in its entirety. In contrast, other studies have focused on endurance sports only or a mixture of different sports, with some of these including only a small number of participants in aesthetic disciplines. Furthermore, responses to open-ended questions enabled the formulation of specific recommendations by the participants themselves, such as the provision for the use of adapted attire.

However, the low response rate may limit the generalizability of the findings. Additionally, the absence of personal interviews may have influenced the respondents’ comprehension of the questions. Although the questionnaire had not been tested for reliability and validity, the questions were formulated in a clear manner using a Likert scale and were based on previous research and pilot testing. Finally, the absence of data regarding the gender of the coaches and pedagogues restricts the ability to further interpret the athletes’ preference to discuss the MC with female coaches.

Further studies are required to build on the findings of this research, particularly to explore how knowledge and communication about the MC are addressed within coaching and pedagogical contexts in aesthetic sports such as rhythmic gymnastics, ballet, and dance. An investigation of communication strategies between coaches and female athletes may facilitate a deeper understanding of how to enhance menstrual health literacy and address potential discomforts. Furthermore, studies that examine the specific role of female versus male coaches in fostering open discussions about the MC and how this influences athlete health and performance are essential. By addressing these gaps, future studies can offer more targeted recommendations for improving menstrual health management within aesthetic sports.

## 5. Conclusions

This study found that most surveyed rhythmic gymnasts, ballerinas, and dancers lacked knowledge about the MC and its influence on physical fitness and performance. Participants also doubted their coaches’ knowledge about the subject. Overall, communication about the MC seemed to be minimal, and coaches/pedagogues were rarely consulted. The gymnasts, ballerinas, and dancers specifically reported discomfort with their attire during menstruation and recommended options for adjustments in their clothing.

## Figures and Tables

**Table 1:** Participants and characteristics.

Demographic Data	Mean (SD)	Minimum–Maximum
Age	20.6 (4.5)	15–39
Height (cm)	167.5 (5.4)	153.0–179.0
Weight (kg)	59.0 (7.3)	42.0–78.0
BMI	21.0 (2.4)	17.0–31.2
**Level of education**	**n**	**%**
Compulsory primary and secondary school	1	0.9
Upper secondary	35	30.2
Vocational studies	22	19.0
Higher education	58	50.0
**Sport/Dance**	**n**	**%**
Rhythmic gymnasts	29	25.0
Classical ballet	29	25.0
Modern/contemporary dance	15	13.0
Jazz dance	35	30.2
Other dance disciplines *	8	7.0
**Number of years as an active gymnast/dancer**	**Mean (SD)**	**Minimum–Maximum**
	10.7 (4.5)	1–25
**Training load (h/week)**	**Mean (SD)**	**Minimum–Maximum**
	22.9 (8.7)	7–40

* HipHop n = 4, commercial dance n = 2, mix of disciplines n = 2.

**Table 2:** Menstrual function and hormonal contraceptive use.

Menstrual History			
**Menarche**	**Yes (n)**	**No (n)**	
	116	0	
**Age of menarche**	**Mean (SD)**	**Min–Max**	
	13.6 (1.6)	9–18 years	
**Normal menstruation**	**Yes n (%)**	**No n (%)**	**Don’t know/** **Remember n (%)**
	72 (62.1)	19 (16.3)	25 (21.6)
**Heavy menstrual bleeding**	**Yes n (%)**	**No n (%)**	
	47 (40.5)	69 (59.5)	
**Degree of bleeding impact on training/performance (scale 0–10)**	**Mean (SD)**		
	4.36 (4.0)		
**Hormonal contraceptive use**	**Yes**	**No**	
Current use	46 (39.7)	70 (60.3)	

**Table 3:** Knowledge and communication about the MC.

Question	Yes n (%)	No n (%)	I Don’t Know n (%)
Monitoring of the MC	60 (51.7)	56 (48.3)	
Engagement in acquiring knowledge about MC’s influence on training and performance	23 (19.8)	93 (80.2)	
Interest in learning more about the MC’s impact on training and performance	95 (81.9)	2 (1.7)	19 (16.4)
Discussion about the MC in relation to training and performance with …*			
(a) Other athletes	51 (44.0)		
(b) Friends	53 (46.0)		
(c) Family	29 (25.0)		
(d) Doctor	16 (14.0)		
(e) Other health personnel (physiotherapist, etc.)	17 (15.0)		
(f) Coach/pedagogue	14 (12.0)		
(g) No one	37 (32.0)		
**Question** **	**agree** ** n (%)	**disagree** ** n (%)	**neither/nor**
Confidence in own knowledge of MC and training and performance	22 (18.9)	73 (62.9)	21 (36.2)
Confidence in coach’s/pedagogue’s knowledge of MC and training and performance	21 (18.1)	45 (38.8)	50 (43.1)
Preference for discussing MC, training, and performance with female coach/pedagogue	105 (90.5)	2 (1.7)	9 (7.8)

* Multiple responses possible. ** Likert scale with five possible answers from totally agree (1) to totally disagree (5). 1 + 2 and 4 + 5 were combined as agree or disagree, respectively.

## Data Availability

The original contributions presented in the study are included in the article/Supplementary Materials, further inquiries can be directed to the corresponding author.

## Supplementary Materials

The following supporting information can be downloaded at: https://www.mdpi.com/article/10.3390/ijerph22010013/s1, Supplementary File: Questionnaire.
