# Supplementary material for: Knowledge and Communication About the Menstrual Cycle Among Rhythmic Gymnasts, Ballerinas, and Dancers
Source: Int J Environ Res Public Health. 2024 Dec 26;22(1):13. doi: 10.3390/ijerph22010013 (PMC11764990; doi:10.3390/ijerph22010013)
Supplement: Supplementary file 1 [file ijerph-22-00013-s001.zip › ijerph-3324666-supplementary.pdf]

PART 1: Background information

1. Age (years)

_____

2. Height (cm)

_____

3. Weight (kg)

_____

4. Level of education

(1) ❍ Compulsory primary and secondary school

(2) ❍ Upper secondary school

(3) ❍ Vocational school / college

(4) ❍ University / College

5. Which sport / dance?

***If you are doing several dance disciplines, please choose/write your main dance discipline.**

(1) ❍ Rhythmic gymnastics (RG)

(2) ❍ Classical ballet

(3) ❍ Modern / contemporary dance

(4) ❍ Jazz dance

(5) ❍ Other dance discipline /style, please specify: _____

6. Are you a full-time gymnast / dancer?

(1) ❍ Yes

(2) ❍ No

6. What do you do beside your sport / dance?

(1) ❍ Full-time job

(5) ❍ Part-time job

(3) ❍ Studies / school

(4) ❍ Other, please specify: _____

7. Number of years as an active gymnast / dancer

_____

8. Training load (hours per week)

_____

9. Previous injury / injuries

Have you ever had an injury that led to absence from training / competition / performance and / or follow-up from health professionals during your career?

(1) ❍ Yes

(2) ❍ No

9. Previous injury / injuries

If yes, where?
You can select multiple choices if you have had several different injuries.

(1) ❑ Ankle / foot

(2) ❑ Calf

(3) ❑ Knee

(4) ❑ Thigh

(5) ❑ Hip / groin

(6) ❑ Buttock / pelvis

(7) ❑ Back (including lower back)

(8) ❑ Neck

(9) ❑ Shoulder

(10) ❑ Stomach

(11) ❑ Arm / wrist / fingers

(12) ❑ Other, please specify: ______

10. Diet / nutrition

a) How often do you eat the following meals during a normal week?

Please put one cross for each meal.

	Never	1-2 times per week	3-4 times per week	5-6 times per week	Every day
Breakfast	(1) ❍	(2) ❍	(3) ❍	(4) ❍	(5) ❍
Lunch	(1) ❍	(2) ❍	(3) ❍	(4) ❍	(5) ❍
Dinner	(1) ❍	(2) ❍	(3) ❍	(4) ❍	(5) ❍
Supper	(1) ❍	(2) ❍	(3) ❍	(4) ❍	(5) ❍
Restitution meal / snack	(1) ❍	(2) ❍	(3) ❍	(4) ❍	(5) ❍

b) How many servings* of dairy products do you eat during a day?

***One serving is one glass of milk, a yoghurt, an ice cream, a slice of bread/crispbread with two slices of cheese etc.**

(1) ❍ 1

(2) ❍ 2

(3) ❍ 3 or more

(4) ❍ Not every day

(5) ❍ I don't eat dairy products

c) How much fruits and vegetables do you eat during a day?

(1) ❍ 1 - 2

(2) ❍ 3 - 4

(3) ❍ 5 or more

(4) ❍ Not every day

(5) ❍ I don't eat fruits and vegetables

d) Do you usually eat or drink before a workout session (up to two hours before)?

(1) ❍ Yes, please specify: _____

(2) ❍ No

e) Do you usually eat or drink during a workout session?

(1) ❍ Yes, please specify: _____

(2) ❍ No

f) Do you usually eat or drink after a workout session?

(1) ❍ Yes, please specify: _____

(2) ❍ No

g) Do you use any supplements?
Eg. vitamins, omega 3 etc.

(1) ❍ Yes

(2) ❍ No

g) What supplements do you use?
You can select multiple choices if you use more than one supplement.

(1) ❑ Cod liver oil

(2) ❑ Omega-3

(3) ❑ Iron

(4) ❑ Vitamin C

(5) ❑ Calcium

(6) ❑ Other, please specify: _____

11. Sleep / restitution

a) How much have you slept on average per night on weekdays* during the last 4 weeks?
(*weekdays = Monday - Friday)

(1) ❍ 5 hours or less

(2) ❍ 6 hours

(4) ❍ 7 hours

(5) ❍ 8 hours

(6) ❍ 9 hours or more

b) How much have you slept on average per night during the weekend* the last 4 weeks?
(*weekend = Saturday and Sunday)

(1) ❍ 5 hours or less

(2) ❍ 6 hours

(4) ❍ 7 hours

(5) ❍ 8 hours

(6) ❍ 9 hours or more

c) Do you sleep during daytime?

(1) ❍ No

(2) ❍ Yes, please specify how many minutes per day: _____

PART 2: Menstruation and the menstrual function (questions from the LEAF-Q)

Mark the response that most accurately describes your situation.

1. Have you ever had a period?
(A single bleed = yes)

(1) ❍ Yes

(2) ❍ No

1. If no, are you undergoing any treatment or measures to initiate menstruation?

(1) ❍ Yes, please explain briefly what kind of treatment/measure: _____

(2) ❍ No

1. If yes, how old were you when you had your first period?

_____

2. Did your first menstruation come naturally (by itself)?

(1) ❍ Yes

(2) ❍ No

(3) ❍ I don't remember

2. If no, what kind of treatment was used to start your menstrual cycle?

(1) ❑ Hormonal treatment

(2) ❑ Weight gain

(3) ❑ Reduced amount of exercise

(4) ❑ Other

3. Do you have normal menstruation?

(1) ❍ Yes

(2) ❍ No

(3) ❍ I don't know

4. When was your last period?

(1) ❍ 0 - 4 weeks ago

(2) ❍ 1 - 2 months ago

(3) ❍ 3 - 4 months ago

(4) ❍ 5 months ago or more

5. Are your periods regular? (every 28th to 34th day)

(1) ❍ Yes, most of the time

(2) ❍ No, mostly not

6. For how many days do you normally bleed??

(1) ❍ 1 - 2 days

(2) ❍ 3 - 4 days

(3) ❍ 5 - 6 days

(4) ❍ 7 - 8 days

(5) ❍ 9 days or more

7. How many periods have you had during the last year?

(1) ❍ 12 or more

(2) ❍ 9 - 11

(3) ❍ 6 - 8

(4) ❍ 3 - 5

(5) ❍ 0 - 2

8. When did you have your last period?

(1) ❍ 2 - 3 months ago

(2) ❍ 4 - 5 months ago

(3) ❍ 6 months ago or more

(4) ❍ I use hormonal contraception and therefore do not menstruate

(5) ❍ I'm pregnant and therefore do not menstruate

9. Have your periods ever stopped for 3 consecutive months or longer (besides pregnancy or hormonal contraception use)?

(1) ❍ No, never

(2) ❍ Yes, it has happened before

(3) ❍ Yes, that's the situation now

10. Do you experience that your menstruation changes when you increase your exercise intensity, frequency or duration?

(1) ❍ Yes

(2) ❍ No

10. If yes, how does your menstruation change when you increase your exercise intensity, frequency or duration? You can check one or more options.

(1) ❑ I bleed less

(2) ❑ I bleed fewer days

(3) ❑ My menstruation stop

(4) ❑ I bleed more

(5) ❑ I bleed more days

11. Have you ever had problems with heavy menstrual bleeding?

(1) ❍ Yes

(2) ❍ No

11. If yes, how much does heavy menstrual bleeding affect you in training and/or competition/performance?

Choose a number between 0 (not at all) and 10 (very much)

(1)	(2)	(3)	(4)	(5)	(6)	(7)	(8)	(9)	(10)	(11)
❍ 0 (not at all)	❍ 1	❍ 2	❍ 3	❍ 4	❍ 5	❍ 6	❍ 7	❍ 8	❍ 9	❍ 10 (very much)

PART 3: Use of hormonal contraceptives

1. Do you currently use any type of hormonal contraceptive or hormonal treatment?

(1) ❍ Yes

(2) ❍ No

2. What type of hormonal contraceptive method or hormonal treatment are you currently using?

(1) ❍ Oral contraceptives, product name: _____

(2) ❍ Mini-pills, product name: _____

(3) ❍ Vaginal-ring, product name: _____

(4) ❍ Injection, product name: _____

(5) ❍ Contraceptive-patch, product name: _____

(6) ❍ Contraceptive implant, product name: _____

(7) ❍ IUD / IUS, product name: _____

(8) ❍ Other, please specify: _____

3. How long have you been using the hormonal contraceptive or the hormonal treatment you are currently using?

Please write number of years and/or months. E.g. 1 year and 3 months or 6 months.

_____

4. Why do you use a hormonal contraceptive or a hormonal treatment?

You can check one or more options.

(1) ❑ To avoid pregnancy

(2) ❑ To reduce menstrual pain

(4) ❑ To reduce menstrual bleeding

(5) ❑ To manipulate the bleeding period

(6) ❑ To have a regular period

(7) ❑ Other reason, please specify: _____

5. Have you experienced that a hormonal contraceptive or a hormonal treatment affect your physical fitness or performance?

(1) ❍ Yes, affects positivley. Please explain briefly how: _____

(2) ❍ Yes, affects negativley. Please explain briefly how: _____

(3) ❍ No influence

(4) ❍ I don't know

6. Have you ever changed or stopped using a hormonal contraceptive or a hormonal treatment?

(Answer yes if you have used something else than what you are using now).

(1) ❍ Yes. Please explain briefly why: _____

(2) ❍ No

6. What type of hormonal contraceptive or hormonal treatment did you use before?

You can check one or more options.

(1) ❑ Oral contraceptives

(2) ❑ Mini-pills

(3) ❑ Vaginal-ring

(4) ❑ Injection

(5) ❑ Contraceptive-patch

(6) ❑ Contraceptive implant

(7) ❑ IUD / IUS

(8) ❑ Other, please specify: _____

PART 4: The menstrual cycle and symptoms

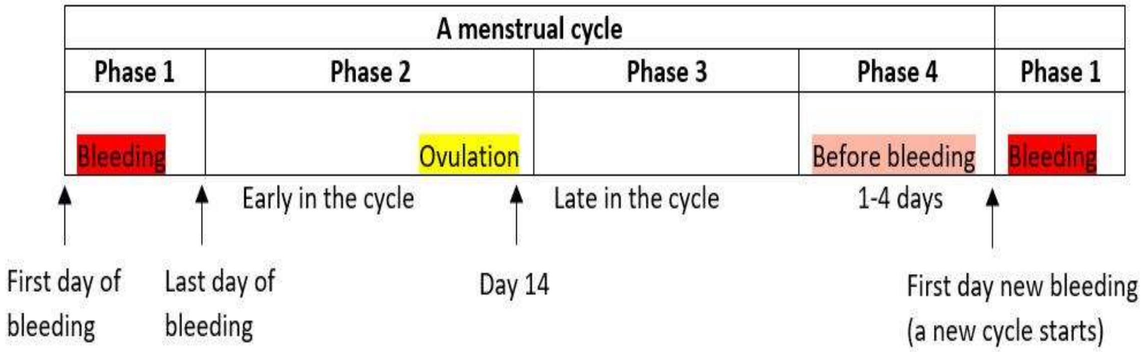

Take some time to look at this picture which defines the different phases of a menstrual cycle (including the start of the next cycle).
In the following pages, you will be asked to answer some questions about **<u>symptoms</u>** related to the different phases of a menstrual cycle (phase 1 - 4).

Phase 1: Bleeding
Phase 2: Early in the cycle, including a possible ovulation
Phase 3: Late in the cycle, after a possible ovulation
Phase 4: The last days before a new bleeding (1-4 days)

Note! Think about <u>the last year</u> when you answer.

1. Do you experience any symptoms during a menstrual cycle?

(1) ❍ Yes

(2) ❍ No

(3) ❍ I don't know

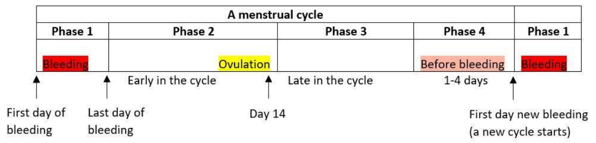

1. If yes, please specify which symptom(s) and when they occur during a menstrual cycle (which phases)?

You can check one or more options.

	Never / not relevant	Phase 1	Phase 2	Phase 3	Phase 4
Pain in the stomach	(5) ❑	(1) ❑	(2) ❑	(3) ❑	(4) ❑
Pain in the lower back	(5) ❑	(1) ❑	(2) ❑	(3) ❑	(4) ❑
Beam pain down the legs	(5) ❑	(1) ❑	(2) ❑	(3) ❑	(4) ❑
Nausea	(5) ❑	(1) ❑	(2) ❑	(3) ❑	(4) ❑
Diarrhoea	(5) ❑	(1) ❑	(2) ❑	(3) ❑	(4) ❑
Constipation	(5) ❑	(1) ❑	(2) ❑	(3) ❑	(4) ❑

Mood swings	(5) ❑	(1) ❑	(2) ❑	(3) ❑	(4) ❑
Bloating	(5) ❑	(1) ❑	(2) ❑	(3) ❑	(4) ❑
Temperature fluctuations	(5) ❑	(1) ❑	(2) ❑	(3) ❑	(4) ❑
Increased appetite	(5) ❑	(1) ❑	(2) ❑	(3) ❑	(4) ❑
Decreased appetite	(5) ❑	(1) ❑	(2) ❑	(3) ❑	(4) ❑
Weight gain	(5) ❑	(1) ❑	(2) ❑	(3) ❑	(4) ❑
Headache / migraine	(5) ❑	(1) ❑	(2) ❑	(3) ❑	(4) ❑
Stomach cramps	(5) ❑	(1) ❑	(2) ❑	(3) ❑	(4) ❑
I feel uncoordinated	(5) ❑	(1) ❑	(2) ❑	(3) ❑	(4) ❑
Bad or blemished skin / acne	(5) ❑	(1) ❑	(2) ❑	(3) ❑	(4) ❑
Low energy / tierdness	(5) ❑	(1) ❑	(2) ❑	(3) ❑	(4) ❑
Other*	(5) ❑	(1) ❑	(2) ❑	(3) ❑	(4) ❑

***Please specify which other symptom:**

_____

2. Have you ever changed your training sessions because of menstrual cycle symptoms?

(1) ❍ Yes

(2) ❍ No

2. If yes, which symptom(s) made you change your training session?

_____

3. Do you use medication to skip/manipulate the bleeding phase?

(1) ❍ Never

(2) ❍ Rarely (1 -3 months per year)

(3) ❍ Sometimes (>3 months per year)

(4) ❍ Often (almost every month)

(5) ❍ Very often (every month)

3. Why do you use medication to skip/manipulate the bleeding phase?

Please explain briefly.

_____

4. Do you use painkillers to reduce menstrual pain?

(1) ❍ Never

(2) ❍ Rarely (1 -3 months per year)

(3) ❍ Sometimes (>3 months per year)

(4) ❍ Often (almost every month)

(5) ❍ Very often (every month)

PART 5: The menstrual cycle, exercise/quality of training and performance

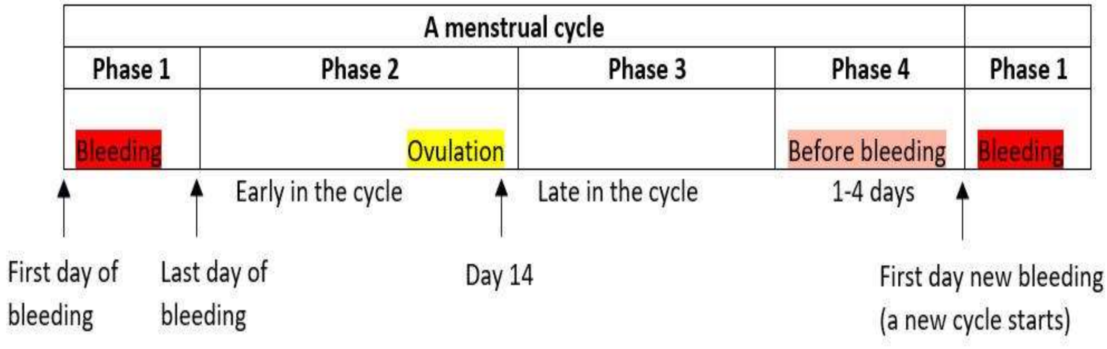

Take some time to look at this picture which defines the different phases of a menstrual cycle (including the start of the next cycle). In the following pages, you will be asked to answer some questions about **exercise/quality of training and performance** related to the different phases of a menstrual cycle (phase 1 - 4)

Phase 1: Bleeding
Phase 2: Early in the cycle, including a possible ovulation
Phase 3: Late in the cycle, after a possible ovulation
Phase 4: The last days before a new bleeding (1-4 days)

Note! Think about the last year when you answer.

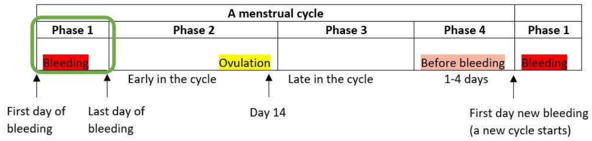

1. How do you experience that the bleeding phase (phase 1) affects the various factors below?

	Very negative	Negative	Neither nor	Positive	Very positive
Performance	(1) ❍	(2) ❍	(3) ❍	(4) ❍	(5) ❍

Physical fitness / quality of training	(1) ❍	(2) ❍	(3) ❍	(4) ❍	(5) ❍
Sleep quality	(1) ❍	(2) ❍	(3) ❍	(4) ❍	(5) ❍
Readiness to train /compete /perform	(1) ❍	(2) ❍	(3) ❍	(4) ❍	(5) ❍
Low intensity training	(1) ❍	(2) ❍	(3) ❍	(4) ❍	(5) ❍
High intensity training	(1) ❍	(2) ❍	(3) ❍	(4) ❍	(5) ❍
Strength training	(1) ❍	(2) ❍	(3) ❍	(4) ❍	(5) ❍

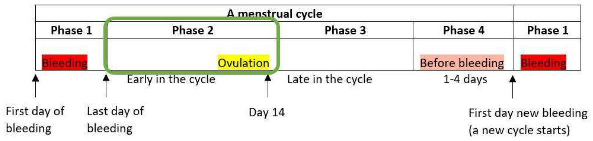

2. How do you experience that the phase early in the cycle (including a possible ovulation), phase 2, affects the various factors below?

	Very negative	Negative	Neither nor	Positive	Very positive
Performance	(1) ❍	(2) ❍	(3) ❍	(4) ❍	(5) ❍
Physical fitness / quality of training	(1) ❍	(2) ❍	(3) ❍	(4) ❍	(5) ❍

Sleep quality	(1) ❍	(2) ❍	(3) ❍	(4) ❍	(5) ❍
Readiness to train /compete /perform	(1) ❍	(2) ❍	(3) ❍	(4) ❍	(5) ❍
Low intensity training	(1) ❍	(2) ❍	(3) ❍	(4) ❍	(5) ❍
High intensity training	(1) ❍	(2) ❍	(3) ❍	(4) ❍	(5) ❍
Strength training	(1) ❍	(2) ❍	(3) ❍	(4) ❍	(5) ❍

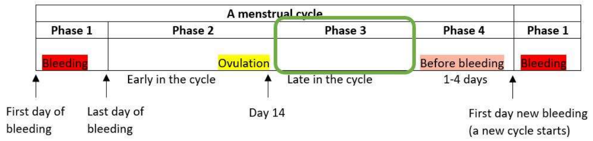

3. How do you experience that the phase late in the cycle (after a possible ovulation), phase 3, affects the various factors below?

	Very negative	Negative	Neither nor	Positive	Very positive
Performance	(1) ❍	(2) ❍	(3) ❍	(4) ❍	(5) ❍
Physical fitness / quality of training	(1) ❍	(2) ❍	(3) ❍	(4) ❍	(5) ❍
Sleep quality	(1) ❍	(2) ❍	(3) ❍	(4) ❍	(5) ❍

Readiness to train /compete /perform	(1) ❍	(2) ❍	(3) ❍	(4) ❍	(5) ❍
Low intensity training	(1) ❍	(2) ❍	(3) ❍	(4) ❍	(5) ❍
High intensity training	(1) ❍	(2) ❍	(3) ❍	(4) ❍	(5) ❍
Strength training	(1) ❍	(2) ❍	(3) ❍	(4) ❍	(5) ❍

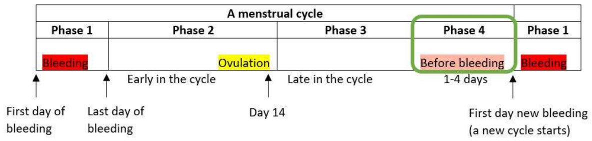

4. How do you experience that the last days before a new bleeding (phase 4) affects the various factors below?

	Very negative	Negative	Neither nor	Positive	Very positive
Performance	(1) ❍	(2) ❍	(3) ❍	(4) ❍	(5) ❍
Physical fitness / quality of training	(1) ❍	(2) ❍	(3) ❍	(4) ❍	(5) ❍
Sleep quality	(1) ❍	(2) ❍	(3) ❍	(4) ❍	(5) ❍
Readiness to train /compete /perform	(1) ❍	(2) ❍	(3) ❍	(4) ❍	(5) ❍

Low intensity training	(1) ❍	(2) ❍	(3) ❍	(4) ❍	(5) ❍
High intensity training	(1) ❍	(2) ❍	(3) ❍	(4) ❍	(5) ❍
Strength training	(1) ❍	(2) ❍	(3) ❍	(4) ❍	(5) ❍

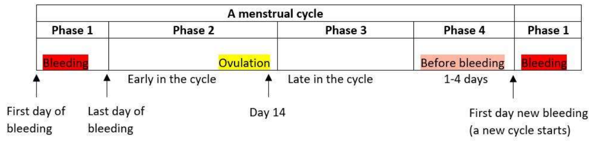

5. In what phase do you experience your best performance?

You can check one or more options.

(1) ❑ Phase 1

(2) ❑ Phase 2

(3) ❑ Phase 3

(4) ❑ Phase 4

(5) ❑ I have not experienced any difference

(6) ❑ I don't know

6. In what phase do you experience your best physical fitness (training quality)?

You can check one or more options.

(1) ❑ Phase 1

(2) ❑ Phase 2

(3) ❑ Phase 3

(4) ❑ Phase 4

(5) ❑ I have not experienced any difference

(6) ❑ I don't know

PART 6: Knowledge and communication

Well done - you have now reached the last part of the questionnaire!

1. Do you monitor/register your menstrual cycle?

(1) ❍ Yes

(2) ❍ No

2. Decide on the following statement:

"I think I have enough knowledge on how the menstrual cycle can affect training and performance."

(1) ❍ Totally agree

(2) ❍ Slightly agree

(3) ❍ Neither nor

(4) ❍ Slightly disagree

(5) ❍ Totally disagree

3. Have you actively engaged in acquiring knowledge on the menstrual cycle, exercise and performance?

(1) ❍ Yes

(2) ❍ No

4. Do you want to learn more about how the menstrual cycle can affect exercise and performance?

(1) ❍ Yes

(2) ❍ No

(3) ❍ I don't know

4. If yes, through which channels* do you think you would have had the best opportunity to learn more about the menstrual cycle, exercise and performance?
***Examples can be webinars, Instagram / Facebook, Google, healthcare professionals, coaches, family and friends, etc.**

Please specify briefly:

____

5. Have you discussed the menstrual cycle in relation to training and performance with others?

You can check one or more options.

(1) ❑ Yes, other athletes

(2) ❑ Yes, friends

(3) ❑ Yes, family

(4) ❑ Yes, doctor

(5) ❑ Yes, other health personnel (physiotherapist etc.)

(6) ❑ Yes, coach/pedagogue/teacher

(7) ❑ No one

6. Decide on the following statement:

"It's easier to talk to a female than a male coach/pedagogue/teacher about topics related to the menstrual cycle, training and performance."

(1) ❍ Totally agree

(2) ❍ Slightly agree

(3) ❍ Neither nor

(4) ❍ Slightly disagree

(5) ❍ Totally disagree

7. Decide on the following statement:

"I think my coach/pedagogue/teacher has enough knowledge about the menstrual cycle in relation to training and performance."

(1) ❍ Totally agree

(2) ❍ Slightly agree

(3) ❍ Neither nor

(4) ❍ Slightly disagree

(5) ❍ Totally disagree

8. Leotard / Suit / Costume

a) Decide on the following statement regarding leotard/costume:

"I have felt uncomfortable during a menstrual bleeding due to the shape and/or color of the leotard/suit/costume in a competition/performance."

(1) ❍ Totally agree

(2) ❍ Slightly agree

(3) ❍ Neither nor

(4) ❍ Slightly disagree

(5) ❍ Totally disagree

b) Do you have any suggestions for changes in shape and/or color that can make the leotard/suit/costume feel less uncomfortable in a competition/performance?

(1) ❍ Yes, please specify breifly: _____

(2) ❍ No

You have now completed the questionnaire!

Do you have any comments to the research group or something you would like to add before the questionnaire ends? (voluntary)

(1) ❍ Yes, please write here: _____

(2) ❍ No

Thank you so much for taking the time to participate in our research project and answer these questions!

Please press "Finish" to complete the survey and submit your answers.

Wish you a nice day!
